# Early childhood predictors of toddlers’ physical activity: longitudinal findings from the Melbourne InFANT Program

**DOI:** 10.1186/1479-5868-10-123

**Published:** 2013-11-05

**Authors:** Jill Hnatiuk, Jo Salmon, Karen J Campbell, Nicola D Ridgers, Kylie D Hesketh

**Affiliations:** 1 Centre for Physical Activity and Nutrition Research, Deakin University, 221 Burwood Highway, Burwood, VIC 3125, Australia

**Keywords:** Early childhood, Physical activity, Toddlers, Infant, Maternal behaviour, Home environment

## Abstract

**Background:**

Young children are at risk of not meeting physical activity recommendations. Identifying factors from the first year of life which influence toddlers’ physical activity levels may help to develop targeted intervention strategies. The purpose of this study was to examine early childhood predictors of toddlers’ physical activity across the domains of maternal beliefs and behaviours, infant behaviours and the home environment.

**Methods:**

Data from 206 toddlers (53% male) participating in the Melbourne InFANT Program were collected in 2008–2010 and analysed in 2012. Mothers completed a survey of physical activity predictors when their child was 4- (T1) and 9- months old (T2). Physical activity was assessed by ActiGraph GT1M accelerometers at 19- months (T3) of age.

**Results:**

One infant behaviour at T1 and one maternal belief and two infant behaviours at T2 showed associations with physical activity at T3 and were included in multivariate analyses. After adjusting for the age at which the child started walking and maternal education, the time spent with babies of a similar age at 4-months (β = 0.06, 95% CI [0.02, 0.10]) and the time spent being physically active with their mother at 9-months (β = 0.06, 95% CI [0.01, 0.12]) predicted children’s physical activity at 19-months of age.

**Conclusions:**

Promotion of peer-interactions and maternal-child co-participation in physical activity could serve as a health promotion strategy to increase physical activity in young children. Future research is required to identify other early life predictors not assessed in this study and to examine whether these factors predict physical activity in later life stages.

## Background

Regular engagement in physical activity has been associated with a number of positive physical and psychosocial health outcomes in early childhood (birth to five years), including decreased body mass index and fatness [1-4], lower cardiovascular risk factors [5,6], improved bone development [7,8], and improved cognitive, social and emotional development [9,10]. However, currently many preschool-aged children do not meet international physical activity recommendations [11] of 180 minutes of light, moderate and vigorous physical activity per day [12-14] and a proportion of younger children (<2 years old) also fail to meet recommendations [15,16]. As such, interventions targeting physical activity promotion among young children have emerged in recent years to increase physical activity levels, although these remain scarce and have demonstrated limited success [17]. Interventions may have greater likelihood of success if they target known predictors of children’s physical activity. While some research exists in preschool-aged children [18,19], no research to date has investigated which factors predict physical activity in younger children.

The Social Cognitive Theory- Family Perspective [20] (SCT-FP) is an extension of the Social Cognitive Theory [21] that highlights the importance of the family on the development of children’s physical activity behaviours. It postulates that bi-directional influences of parent and child beliefs and behaviours as well as the home environment interact to affect children’s physical activity. This model is particularly relevant for understanding young children’s physical activity given their unique dependence on and close relationship with parents, often the child’s mother. The SCT-FP model highlights potential constructs (i.e. maternal beliefs, maternal behaviours, infant behaviours, home environment) from which potential early predictor variables can be identified to inform future intervention strategies. Therefore, the aim of this paper was to examine early childhood predictors of young children’s physical activity across the domains of maternal beliefs and behaviours, infant behaviours and the home environment.

## Methods

### Participants

Participant data were obtained from the Melbourne Infant Feeding, Activity & Nutrition Trial (InFANT) Program. Ethics approval for the study was obtained from the Deakin University Ethics Committee and from the Victorian Government’s Office for Children and parents provided informed consent for their own and their child’s participation. The rationale and methods used in the Melbourne InFANT Program have been described in detail previously [15,22,23]. Briefly, the Melbourne InFANT Program was a low-dose cluster randomized controlled trial that aimed to provide first-time parents with the skills, knowledge and confidence to reduce obesity-promoting behaviours, including physical inactivity. Parents were recruited through first time parent groups operated by the free, universal maternal and child health centres in randomly selected Local Government Areas. The 15-month intervention was conducted with 542 parent–child pairs from 62 different parent groups between June 2008 and February 2010 when participating children were between 4- and 19-months of age. Data were analysed in 2012.

### Procedures

Questionnaires assessing all predictor variables were self-completed by mothers when the children were aged 4-months (T1- baseline) and 9-months (T2- mid-intervention). Physical activity was assessed every 15 seconds for a minimum of 7 consecutive days using ActiGraph GT1M accelerometers, removed only for sleeping and bathing, when the children were approximately 19-months old (T3- intervention conclusion). As there were no differences in total physical activity between intervention and control group children at intervention conclusion (225.3 ± 41.0 mins vs. 237.4 ± 38.6 mins, p > 0.19) all data were pooled for this study. There were also no intervention group differences for the predictor variables with the exception that mothers’ physical activity at T1 was higher in the control group (removal of this item did not impact results, hence it was retained).

### Measures

#### ***Participant demographics***

The child’s sex and date of birth, and maternal education were assessed through the mother’s questionnaire at T1. Maternal education was categorized as low (secondary school or less), middle (trade and certificate qualifications) or high (university degree or higher), consistent with previous research [24]. Maternal employment status was assessed at each time point and categorized as: on maternity leave/home duties full time, full-time employment, part-time employment or other (consisting of student, retired, unemployed, other). The age that the child started walking was retrospectively asked in the mother’s questionnaire at T3. The time since the child began walking was calculated by subtracting the age when the child began walking from the child’s age when the mother completed the questionnaires.

### Predictor variables

#### ***Maternal beliefs***

Maternal beliefs were assessed at T1 (child 4-months old) through 36 purpose-designed survey questions covering maternal beliefs, attitudes and intentions regarding their child’s physical activity and television viewing. All questions were scored on a 4-point likert-type scale (0 = strongly disagree/not at all confident to 3 = strongly agree/extremely confident). The questions were based on previous qualitative [25] and quantitative [26] work and were tested in a separate sample of parents of infants with moderate to good test-retest reliability (% agreement = 0.56-0.86). Nine factors were generated from the 36 items using exploratory factor analyses with promax rotation in the larger study sample: physical activity knowledge (10 questions examining the importance of physical activity for babies’ and toddlers’ health and development), views of physically active children (4 questions examining maternal perceptions of active children), physical activity optimism (3 questions examining the anticipated ease of engaging children in physical activity), self-efficacy for promoting physical activity (3 questions examining mothers’ confidence for promoting physical activity), future expectations around children’s physical activity and TV viewing (2 questions examining maternal expectations of children’s future physical activity and TV viewing behaviours), floor play concerns (2 questions examining perceptions of safety of floor play), TV knowledge (4 questions examining perceived benefits of TV for young children), TV use (5 questions examining the use of TV for practical reasons) and self-efficacy for limiting TV (3 questions examining mothers’ confidence for limiting TV viewing). Factor scores were generated by averaging the item scores within each factor. All factors at T1 had good internal reliability in the larger sample (Chronbach’s α = 0.58–0.87) and in the subsample for this paper (Cronbach’s α = 0.54-0.88).

At T2, mothers completed a shortened version of the T1 questionnaire focused on main outcomes (to reduce participant burden). The T2 questionnaire contained five of the nine original maternal belief factors: physical activity optimism, self-efficacy for promoting physical activity, future expectations around children’s physical activity and TV viewing, TV use and self-efficacy for limiting TV. Internal reliability for the T2 factors ranged from α = 0.64–0.92 in the subsample for this paper.

#### ***Maternal behaviours***

Mothers’ physical activity was assessed at T1 and T2 using the validated Active Australia Survey [27]. Total physical activity (mins/week) was determined by summing the time spent walking (>10 minutes), the time spent in moderate intensity physical activity (MPA) and twice the time spent in vigorous intensity physical activity (VPA) in the past week, since VPA confers greater health benefits [27]. To avoid errors in over reporting, minutes spent in any given activity intensity were truncated at 840 minutes/week (14 hours) and time spent in all activity intensities was truncated at 1680 minutes/week (28 hours) [27].

Maternal television viewing (TV) time (mins/week) was assessed on weekdays and weekends by the questions, “On a usual weekday (weekend day), about how many hours do you usually spend sitting down and watching television or videos/DVDs?”, shown in previous studies to be valid and reliable [28]. TV time was determined by calculating a weighted average between the weekday and weekend day responses. To avoid errors in over-reporting, total TV times reported were truncated at 1060 minutes/day (18 hours).

#### ***Infant behaviours***

Infant behaviours were assessed at T1 and T2 by maternal proxy-report of the amount of time in the last week the infant spent in a variety of physical activity behaviours. Items included: time spent playing games with an adult, time spent being physically active with mum, time spent on the child’s stomach (tummy time), time spent on the floor, time spent with other babies of a similar age, time spent with older toddlers or children and time spent outside. All infant behaviours were presented as mins/week. Test-retest reliability (ICC) for these items, conducted in the separate sample, ranged from 0.25-0.59 at T1 and 0.41–0.86 at T2.

#### ***Home environment***

At T1 and T2, mothers were asked, on a 4-point likert-type scale (0 = unlikely to 3 = extremely likely), how likely it is that they will provide a variety of activity equipment (appropriate for young children) in their home (e.g., balls, push-along toy, sand pit). Responses were categorised as 'likely’ (score of 1) if the mother responded she 'already had’ or was 'extremely likely’ or 'very likely’ to provide that piece of equipment in their home for their child. Responses were categorized as 'unlikely’ (score of 0) if the mother responded 'possibly’ or 'unlikely’ to provide that piece of equipment in their home for their child. A total score was then calculated by summing the points for the 12 equipment items. At T1 only, mothers were asked, “How many TVs do you have in your home?” Test-retest reliability in the separate sample was fair to good for provision of activity equipment (ICC = 0.49–0.80 at T1 and ICC = 0.48–0.77 at T2) and excellent for the number of TV’s in the home (% agreement = 0.96).

### Accelerometer data reduction

Following current physical activity recommendations, [12-14] the outcome variable was the total time spent in light-to-vigorous intensity physical activity per day. Validated cut-points of 192–1672 and >1672 counts per minute [29] were applied to the data to determine time spent in light- and moderate- to- vigorous intensity physical activity, respectively and then summed to determine total time (mins/day) spent in physical activity for each child. Twenty minutes of consecutive zero counts were identified as non-wear time and only children with at least 4 days (including at least one weekend day) of valid (≥ 7.4 hours/day) data were included in the analyses [15]. As higher physical activity levels were observed on weekends compared with weekdays (240.8 ± 50.8 mins vs. 230.6 ± 42.55 mins) a weighted weekly score was calculated. Active time for each valid day (total activity divided by number of days) were separated into weekday (Monday-Friday), weekend (Saturday, Sunday), and weighted whole week data, where the weekdays provided 5/7ths of weekly activity and the weekends provided 2/7ths.

#### ***Data analyses***

Linear regression analyses were conducted for all predictors (factors and individual items) assessed at T1 (4-months) and T2 (9-months) with child’s physical activity at 19-months as the outcome variable. All models included the following confounder variables: (1) intervention arm (although there was no intervention effect on the outcome variable (physical activity), this was undertaken as a precautionary measure); (2) clustering by the unit of randomization (mother’s group attended; individuals within the same unit are expected to be more alike than if randomly chosen) [30] and (3) average accelerometer wear minutes/day (physical activity level was positively associated with accelerometer wear time in our sample [r = 0.48]). Any predictor which had a p-value of ≤0.10 in Model A analyses was included in Models B and C. Model C also controlled for maternal education which is a known covariate of older children’s physical activity [31,32] and the age that the child began walking, as children who began walking earlier had higher physical activity levels than those who began walking later in this sample (β = -4.30, 95% CI [-6.69, -1.90]). As few mothers were employed at T1, data were also analysed with maternal employment status at T2 and T3 as a covariate, however no differences in results were observed and thus the results are not presented. Variance Inflation Factors in the regression models were all <10 indicating that multicollinearity was not a concern [33]. Analyses were conducted in Stata version 12.0.

## Results

### Participant characteristics

From the 542 parent–child pairs in the Melbourne InFANT Program, 417 children provided accelerometer data at T3, however 130 of these did not have sufficient accelerometer data and a further 81 did not have complete questionnaire data. This resulted in a sample of 206 children (across 60 parent groups) that were included in these analyses. The participant characteristics of this sample are provided in Table 1. Children included in this study were significantly younger, had been walking for less time and had mothers with higher levels of education than children excluded from the study.

**Table 1:** Participant characteristics

**Participant characteristic**	**N = 206**
Male child (%)	53.4
Child age (months): mean (SD)	
T1	3.5 (1.0)
T2	8.8 (1.0)
T3	18.7 (2.0)
Age child began walking (months): mean (SD)	13.1 (1.8)
Time since child began walking (months): mean (SD)	5.6 (2.6)
Total light-vigorous physical activity (mins/day): mean (SD)	233.5 (41.0)
Maternal education^a^ (%)	
Low	12.1
Medium	26.2
High	61.7

^a^Low = secondary school or less; Medium = trade or certificate qualifications; High = university degree or higher.

### Early predictors of toddlers’ physical activity

Table 2 displays the mean scores for all predictor variables at T1 & T2. Table 3 presents the results of the linear regression models from the T1 predictor items and factors. One infant behaviour item (time spent with other babies of a similar age) had a p-value of <0.10 and was included in Model B and C analyses. In both Models B and C, time spent with other babies of a similar age remained positively associated with toddlers’ PA. In Model B, 22.8% of the variance was accounted for by the included variables (22.5% from covariates). In Model C (which also included maternal education and age at walking), 27.2% of the variance was accounted for by the included variables (26.7% from covariates).

**Table 2:** **Differences in predictor variables between T1** (**child aged 4**-**months**) **and T2** (**child aged 9**-**months**)

**Variable**	**T1**	**T2**	**p**-**value**^**c**^
	**Mean (SD)**	**Mean (SD)**	
**Maternal beliefs**^**a**^			
Physical activity knowledge^b^	2.52 (0.33)	-	-
Physical activity views^b^	0.87 (0.45)	-	-
Physical activity optimism	2.33 (0.44)	2.49 (0.46)	**0.00**
Physical activity self-efficacy	2.53 (0.50)	2.57 (0.49)	0.23
Future expectations	1.74 (0.50)	1.81 (0.58)	**0.02**
Floor concerns^b^	0.93 (0.55)	-	-
TV knowledge^b^	1.61 (0.53)	-	-
TV use	0.74 (0.45)	0.65 (0.64)	**0.02**
TV self-efficacy	2.02 (0.63)	2.16 (0.63)	**0.00**
**Maternal behaviours**			
Maternal physical activity (mins/week)	485.19 (380.24)	501.28 (386.75)	0.67
Maternal screen time (mins/week)	221.79 (140.31)	162.36 (124.31)	**0.00**
**Infant behaviours**			
Time spent playing games with adult (mins/week)	58.71 (59.80)	67.75 (59.34)	0.08
Time spent being physically active with mum (mins/week)	62.12 (59.39)	73.83 (64.51)	**0.03**
Time spent having tummy time (mins/week)	25.99 (34.20)	81.50 (97.31)	**0.00**
Time spent on the floor (mins/week)	97.27 (82.14)	206.59 (117.20)	**0.00**
Time spent with other babies of a similar age (mins/week)	27.18 (50.34)	52.26 (143.25)	**0.01**
Time spent with older toddlers or children (mins/week)	20.00 (45.65)	33.63 (53.48)	**0.01**
Time spent outside (mins/week)	46.93 (46.64)	61.10 (48.35)	**0.00**
**Home environment**			
Physical activity equipment in home (# items)	6.31 (2.27)	5.67 (1.22)	**0.00**
TVs in home (# TVs)^b^	1.87 (0.93)	-	-

^a^Scored on a scale of 0–3 where a higher score reflects greater agreement with the belief.

^b^Not assessed at T2.

^c^p-values <0.05 are bold.

**Table 3:** **Early childhood predictors** (**T1**- **4**-**months of age**) **of toddlers**’ **physical activity** (**mins**/**day**) **at 19**-**months of age**

**Variable**	**Model A**^**a**^	**Model B**^**b**^	**Model C**^**c**^
	**β (95% CI)**	**β (95% CI)**	**β (95% CI)**
**Maternal beliefs**			
Physical activity knowledge	2.76 (-16.01, 21.52)		
Physical activity views	-0.73 (-10.76, 9.30)		
Physical activity optimism	8.19 (-4.49, 20.88)		
Physical activity self-efficacy	0.30 (-10.80, 11.39)		
Future expectations	5.51 (-4.51, 15.54)		
Floor concerns	-4.17 (-13.24, 4.88)		
TV knowledge	6.95 (-3.76, 17.66)		
TV use	4.95 (-6.93, 16.83)		
TV self-efficacy	1.70 (-7.13, 10.52)		
**Maternal behaviours**			
Maternal physical activity (mins/week)	0.01 (-0.01, 0.02)		
Maternal screen time (mins/week)	-0.01 (-0.04, 0.01)		
**Infant behaviours**			
Time spent playing games with adult (mins/week)	-0.01 (-0.08, 0.07)		
Time spent being physically active with mum (mins/week)	0.01 (-0.06, 0.08)		
Time spent having tummy time (mins/week)	0.02 (-0.12, 0.15)		
Time spent on the floor (mins/week)	0.02 (-0.04, 0.08)		
Time spent with other babies of a similar age (mins/week)	0.04 (0.01, 0.08)	0.04 (0.01, 0.08)	0.06 (0.02, 0.10)
Time spent with older toddlers or children (mins/week)	-0.02 (-0.11, 0.07)		
Time spent outside (mins/week)	0.01 (-0.10, 0.11)		
**Home environment**			
Physical activity equipment in home (# items)	0.09 (-2.26, 2.44)		
TVs in home (# TVs)	0.43 (-4.35, 5.22)		

^a^Adjusted for accelerometer wear time, intervention group and clustering by mother’s group attended.

^b^Adjusted for accelerometer wear time, intervention group, clustering by mother’s group attended, and all variables associated in Model A (p-value of ≤0.10).

^c^Adjusted for accelerometer wear time, intervention group, clustering by mother’s group attended, all variables associated with PA in Model A (p-value of ≤ 0.10), maternal education and age when the child began walking.

Table 4 presents the results of the linear regression models using the T2 predictor variables. From Model A results, one maternal belief factor (physical activity optimism) and two infant behavioural items (time spent being physically active with mum and time spent with other babies of a similar age) had p-values of <0.10 and were included in Models B and C. In Model B, the time spent with other babies of a similar age was negatively associated with toddlers’ PA and time spent being physically active with mom was positively associated with toddlers’ PA. After controlling for age when the child began walking and maternal education (Model C) only the time spent being physically active with mum remained associated with toddlers’ PA. In Model B, 27.7% of the variance was accounted for by the included variables (22.5% from covariates). In Model C, 30.0% of the variance was accounted for by the included variables (26.7% from covariates).

**Table 4:** **Early childhood predictors** (**T2**- **9**-**months of age**) **of toddlers**’ **physical activity** (**mins**/**day**) **at 19**-**months of age**

**Variable**	**Model A**^**a**^	**Model B**^**b**^	**Model C**^**c**^
	**β (95% CI)**	**β (95% CI)**	**β (95% CI)**
**Maternal beliefs**			
Physical activity optimism	10.83 (-1.16, 22.82)	10.21 (-1.75, 22.16)	9.24 (-2.34, 20.81)
Physical activity self-efficacy	6.03 (-6.01, 18.06)		
Future expectations	6.31 (-2.65, 15.28)		
TV use	-3.03 (-11.38, 5.32)		
TV self-efficacy	3.19 (-4.83, 11.20)		
**Maternal behaviours**			
Maternal physical activity (mins/week)	0.01 (-0.01, 0.02)		
Maternal screen time (mins/week)	-0.01 (-0.06, 0.03)		
**Infant behaviours**			
Time spent playing games with adult (mins/week)	0.04 (-0.05, 0.13)		
Time spent being physically active with mum (mins/week)	0.09 (0.02, 0.15)	0.07 (0.01, 0.14)	0.06 (0.01, 0.12)
Time spent having tummy time (mins/week)	-0.03 (-0.07, 0.01)		
Time spent on the floor (mins/week)	0.03 (-0.02, 0.07)		
Time spent with other babies of a similar age (mins/week)	-0.05 (-0.08, -0.01)	-0.04 (-0.08, -0.01)	-0.03 (-0.08, 0.01)
Time spent with older toddlers or children (mins/week)	-0.02 (-0.11, 0.08)		
Time spent outside (mins/week)	0.03 (-0.08, 0.14)		
**Home environment**			
Physical activity equipment in home (# items)	0.43 (-3.79, 4.65)		

^a^Adjusted for accelerometer wear time, intervention group and clustering by mother’s group attended.

^b^Adjusted for accelerometer wear time, intervention group, clustering by mother’s group attended, and all variables associated in Model A (p-value of ≤0.10).

^c^Adjusted for accelerometer wear time, intervention group, clustering by mother’s group attended, all variables associated with PA in Model A (p-value of ≤ 0.10), maternal education and age when the child began walking.

## Discussion

This study was the first to examine early childhood predictors of toddlers’ physical activity at two different time points (4-months and 9-months old). The results indicated that two of the investigated variables, the time spent with other babies of a similar age at child aged 4-months and the time spent being physically active with the child’s mother at child aged 9-months, significantly predicted toddlers’ objectively assessed physical activity, after adjusting for the age the child started walking and maternal education. These findings potentially highlight the importance of social interaction with peers and maternal-child co-participation in physical activity from a young age for children’s physical activity levels.

Previous cross-sectional research has found that 2-year old children’s outdoor play time (used as a proxy for physical activity) was marginally associated with their mother’s self-reported physical activity level [34] and that preschool children’s objectively assessed physical activity is related to mothers’ physical activity levels [35,36] and the frequency that she is active with her child [19]. The findings from the current study suggest that maternal-child co-participation in physical activity early in life may increase children’s physical activity levels as toddlers, although further research is required to better understand this relationship in the early childhood period. The finding that time spent with other children of a similar age at child aged 4-months is more difficult to explain as the positive association between these variables did not persist when assessed at 9-months of age. It is possible that this variable served as a proxy for some other construct at child aged 4-months that was not measured in the study; for example, maternal sociability. While it could be hypothesized that the time children spent with other babies of a similar age was influenced by whether or not they attended a childcare facility, no between-group differences in time spent with other babies of a similar age were observed when assessed via an independent samples t-test (results not shown). However, this insignificant finding may have also occurred due to the fact that very few children (<3%) attended a childcare facility at T1. Future research should further investigate the influence of the time spent with other babies of a similar age and the time spent being physically active with their mother on young children’s physical activity levels before any conclusions can be made.

Although positive associations were observed between children’s physical activity and both the time spent with other peers and being active with their mother, similar findings were not observed in relation to the time spent with older toddlers or children. This is contrary to previous research in school-aged children which suggests that children with an older sibling are more active than children without an older sibling [37]. One possible explanation for this finding is that the children in this study were first born, and thus many of them did not spend any time with older toddlers or children. Having such a high percentage of children who spent no time with older toddlers or children may have reduced the discriminant ability of this variable, making it difficult to determine its influence on physical activity in this cohort.

While only considered as a covariate, this study found that the age when the child began walking was related to their physical activity levels as toddlers. This information is important, as it may serve as a way to identify children at risk of low levels of physical activity, who may benefit from early support strategies to engage in sufficient physical activity. To put the effect size identified in this study into context, there would be a difference of approximately 40 minutes of physical activity per day at 19-months in favour of early walkers (8-months), compared to those who began walking at the uppermost (18-months) limit of the age range of typical motor development for healthy children [38]. Given there is evidence that low levels of physical activity can track into childhood [39], developing strategies from an early age to increase physical activity in children who are at risk may be beneficial. We are aware of only one other study in this area. This work found that certain motor milestones such as age at standing unaided and walking with support were positively associated with a modest increase in sport participation at age 14-years [40]. It is reasonable to assume that the relationship between children’s age at walking and physical activity level may be explained by the length of time that the children had been walking. In other words those with higher levels of physical activity had simply been walking longer and were therefore more adept at upright movement than those who had more recently mastered the skill. However, when the time since walking commenced was examined as a potential predictor of physical activity, no association was observed (results not shown). Future research should examining physical activity levels in young children should account for the age that children started walking in the analyses. Research is also warranted to specifically investigate how motor development is related to future physical activity levels in children.

Despite assessing a range of variables covering the key aspects of the SCT-FP model, most of the variables examined when the children were 4- and 9-months old were not associated with toddlers’ physical activity, suggesting that it is very challenging to identify key early life predictors of physical activity. This finding is consistent with other recent research whereby no modifiable correlates of toddlers’ physical activity were identified [16]. It could be hypothesized that physical activity in young children is largely biologically determined, with external influences having a smaller effect on physical activity compared to older children, at least in the short-term. The finding that the age of walking was significantly associated with objectively assessed physical activity at 19-months lends credence to this hypothesis. Given that this was the first study of its kind, and few studies have reported objectively measured physical activity levels of children under 2-years old, these findings provide a platform to inform subsequent research to develop our understanding of young children’s physical activity behaviour and to identify other potential predictors of physical activity. However, limitations of the manuscript must be acknowledged. As the effect sizes of both the significant variables were small, they were single item variables, and multiple statistical tests were performed, it is possible that the findings observed were due to chance. Additionally, attending a child care facility may have been an important correlate at T2, but this information was not collected. However, attempts were made to account for this limitation by running the regression models with maternal employment status included. Finally, our sample was highly educated and had children who were younger and had been walking for less time. This may preclude generalizability to the wider population.

## Conclusions

The time spent with other babies of a similar age at 4-months of age and the time spent being physically active with the child's mother at 9-months of age were positively (albeit weakly) associated with physical activity levels at 19-months of age. Thus, promotion of peer-interactions and maternal-child co-participation in physical activity could potentially serve as a health promotion strategy to increase physical activity in young children. However, further investigation is required to determine if the time spent with other babies of a similar age at 4-months is associated with toddlers’ physical activity in separate samples, and whether the time spent with other babies of a similar age and time spent being physically active with the child’s mother predicts physical activity in later life stages (e.g. preschool and primary school years). Furthermore, future research should examine other potential early predictors of physical activity not assessed in this study.

## Competing interest

The authors declare that they have no competing interest.

## Authors’ contributions

KC, KH and JS designed the Melbourne InFANT Program from which the data were drawn. JH analysed the data and drafted the article. KH, JS, KC and NR assisted with data analysis and interpretation and revised the manuscript for important intellectual content. All authors conceptualized and designed the idea for the paper and gave approval for the final version to be published.
